# Expression of Progenitor Cell Markers in the Glial-Like Cells of Epiretinal Membranes of Different Origins

**DOI:** 10.1155/2018/7096326

**Published:** 2018-12-27

**Authors:** Xiaohe Yan, Pål Andresen, Xhevat Lumi, Qingshan Chen, Goran Petrovski

**Affiliations:** ^1^Shenzhen Key Laboratory of Ophthalmology, Shenzhen Eye Hospital, Jinan University, Shenzhen 518040, China; ^2^School of Optometry, Shenzhen University, Shenzhen 518040, China; ^3^Center for Eye Research, Department of Ophthalmology, Oslo University Hospital and University of Oslo, Kirkeveien 166, N-0407 Oslo, Norway; ^4^Department of Ophthalmology, University Medical Centre Ljubljana, Ljubljana, Slovenia

## Abstract

**Purpose:**

To investigate the expression of progenitor cell markers (Sox2, Nestin, and Pax2) in idiopathic epiretinal membranes (iERMs) and nonidiopathic epiretinal membranes (niERMs) in relation to glial cell marker expression.

**Methods:**

ERMs were obtained from patients with iERMs and niERMs of different origins: proliferative diabetic retinopathy (PDR), proliferative vitreoretinopathy (PVR), and uveitis. The membranes were studied by flat-mount or sectional immunohistochemistry for expression of progenitor cell markers as well as glial (GFAP) and proliferation (Ki-67) markers.

**Results:**

Cells in the ERMs express strong GFAP, with strong Pax2 expression in the cell nuclei. Some of the GFAP-positive glial cells in all epiretinal membrane types colocalized with Sox2, Pax2, and Nestin. NiERMs are much more cellular than iERMs. Glial cells are more densely packed in all analyzed niERMs, whereas glial cells with long branches are found in the internal limiting membrane parts and the iERMs, which appear to form a local network by their processes.

**Conclusion:**

The GFAP-positive glial cells in ERMs are not pure glial cells, and some of them express progenitor cell markers, which indicate that these cells may have potential for self-renewal and differentiation into more glial or neuroglial type of cells.

## 1. Introduction

Gliosis is a process of scar formation caused by activated glial cells in the neural system. It is characterized by an upregulated expression of the intermediate filament protein, glial fibrillary acidic protein (GFAP), in glial cells [1]. Epiretinal membranes (ERMs) are a thin sheet of glial scar tissue, which is found on the surface of a pathologically changed retina, as may occur in proliferative diabetic retinopathy (PDR), proliferative vitreoretinopathy (PVR) after retinal detachment surgery, and ocular inflammation/uveitis. ERMs in the human macula may occur without any obvious causes, which is then called idiopathic retinal gliosis or idiopathic ERMs (iERMs). Both the iERMs and nonidiopathic (niERMs) often cause reduced vision and metamorphopsia, which may worsen over time. The exact cause, initial events, progression, and recurrent reasons for formation of ERMs remain largely unknown, as well as the partial use in surgery without regard, as in the internal limiting membrane (ILM) flap technique [2, 3].

Histopathological studies have shown glial cells to be the main cell type in ERMs, which were initially based upon observation of 8 enucleated eyes with ERMs; it appeared the glial cells migrate out of the retina through a ruptured ILM and contribute to ERM formation [4]. Subsequent studies of electron microscopy showed that the cell components of the ERMs are not only glial but also other cell types such as fibroblasts, myofibroblasts, and macrophages [5, 6]. Growth factors may contribute to the pathogenesis of ERMs. The expression of transforming growth factor-beta 2 (TGF-*β*2) and nerve growth factor (NGF) has been found elevated in the vitreous in patients having iERMs [7]. In addition, extracellular matrix molecules such as collagen have also been expressed in ERMs [8]. Interestingly, Nestin, a marker for neural stem cells, has also been detected in ERMs [9]. In addition, glial cells from the vitreous of patients with diabetic retinopathy have been found to coexpress the neural stem/progenitor-cell marker Nestin, and these cells were able to detach from the proliferating ERMs and enter into the vitreous [10]. In the injury-induced mouse brain gliosis, the astrocytes are activated and can proliferate, thus acquire multipotency and properties for self-renewal *in vitro* [11]. The latter study showed that such glial cells found in the gliosis region may contain stem cell properties in order to maintain self-renewal and produce more glial cells. These results indicate that the cells in the ERMs may also not be pure glial cells, and the properties of the ERM-composing cells may be different.

Little is known about the expression of progenitor cell markers (Sox2, Nestin, and Pax2) in human ERMs and ILMs. In this study, we studied the expression of such progenitor cell markers using the postsurgical flat-mount and sectioning immunohistochemistry technique. Moreover, the colocalization of GFAP and proliferation markers in the different types of ERMs is hereby shown. The study has relevance for the future decision-making in surgery, whether to fully or partially discard parts of the ERMs and/or ILMs, which may be a source of progenitor cells other than glial cells.

## 2. Materials and Methods

### 2.1. Ethics and Collection of Materials

The study was approved by the Medical Ethics Committee of University Medical Centre Ljubljana, Slovenia, and informed consent was obtained from each patient according to the tenets of the Declaration of Helsinki. All the ERMs and ILMs were surgically removed by peeling during 23G pars plana vitrectomy from patients with idiopathic and nonidiopathic retinal gliosis (associated with eye diseases) in the Department of Ophthalmology, University Medical Centre Ljubljana. The ERMs samples were collected from 15 patients with retinal gliosis, consisting of 6 patients with secondary retinal gliosis (PDR, PVR, and uveitis) and 9 patients with idiopathic retinal gliosis. All the samples were fixed in 4% PFA for at least 24 hours before further processing.

### 2.2. Immunohistochemistry

For the flat-mount immunohistochemistry analysis, fixed ERMs were rinsed with phosphate-buffered saline (PBS)/0.1%tritonX-100 for two times with each for 10 minutes, and then penetrated in 0.1 M glycin/PBS for 10 minutes. The ERMs were blocked in 3% fetal bovine serum (FBS)/0.1%tritonX-100 for 1 hour at room temperature, and then incubated in primary antibodies against GFAP (rabbit IgG, 1 : 500 dilution, G9269, Sigma-Aldrich), Sox2 (goat IgG, 1 : 500 dilution, No. sc-17320, Santa Cruz), Nestin (mouse IgG, 1 : 500 dilution, No. 561230, BD Pharmingen), and Pax2 (rabbit IgG, 1 : 200 dilution, No. 2549-1, Epitomics) in FBS/0.1%tritonX-100 or blocking solution (Control) for 24 hours at 4°C with shaking at 400 rpm, followed by washing in PBST and then incubation with secondary antibodies with Alexa Fluor 488 (antirabbit, 1 : 250 dilution, No. A21206, Invitrogen) or Cy3 (antigoat, 1 : 250 dilution, No. 705-165-147, Dianova) or Cy5 (antimouse, 1 : 250 dilution, No. 715-175-150, Jackson Immuno) for 1 hr at room temperature. Finally, the nuclei of the samples were stained with 4′,6-diamidino-2-phenylindole (DAPI, 1 : 10000 dilution) (No. D9564, Sigma-Aldrich) for 10 minutes. Supplementary Figure 1 contains the negative controls for all the antibodies/stainings used in the study.

For cryosectional immunohistochemistry, fixed ERM samples were embedded in Tissue-Tek O.C.T. Compound (Sakura Finetek, USA) and stored at −80°C before further processing. ERMs were cut at 12 *μ*m using a cryostat (Leica Microsystems) at −20°C, and a series of sections were collected using SuperFrost® Plus slides (Menzel, Germany). Further immunohistochemical procedures were the same as for the flat-mount immunohistochemistry shown above.

### 2.3. Image Acquisition and Statistical Analysis

Confocal laser scanning microscope (Olympus, Hamburg, Germany) was used to acquire the immunofluorescent images of the samples (single plane images and Z-stacks) and analyzed by FluoView software (Olympus).

## 3. Results

**Figure 1:**
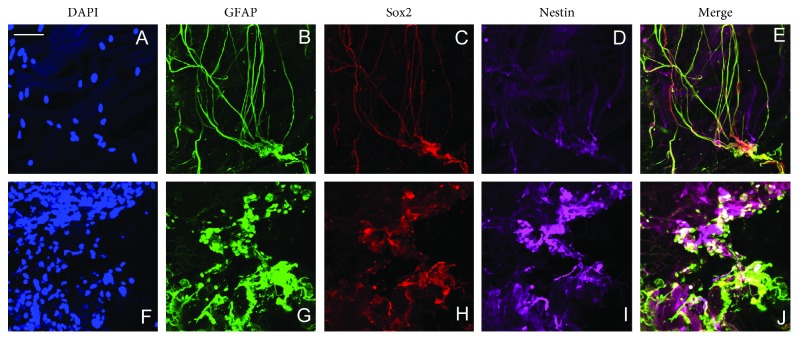
Presence of glial-like cells in the epiretinal membranes from patients with idiopathic retinal gliosis and from patients with nonidiopathic retinal gliosis. Expression of Sox2 and Nestin is also shown in the idiopathic epiretinal membranes (a–e) and in the nonidiopathic retinal gliosis (from PVR) (f–j). Scale bar: 20 *µ*m.

In both iERMs and all types of niERMs, GFAP was highly and densely expressed, suggesting these cells are activated glial cells (Figures 1(b) and 1(g)). These cells form long processes extending from the cell bodies, which appear to form a local network of cells within the ERMs from patients with idiopathic retinal gliosis (Figures 1(a) and 1(b)). The activated glial cells coexpress stem cell markers Sox2 and Nestin (Figures 1(a)–1(e)). Some of the cell processes appear to coexpress Sox2, but not Nestin, while other processes only express Nestin, but not Sox2 or GFAP.

**Figure 2:**
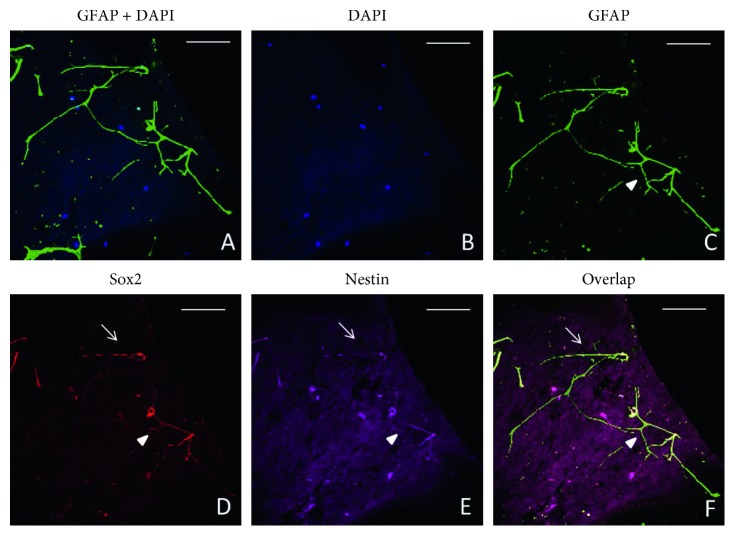
Presence of glial-like cells in the inner limiting membrane from patients with idiopathic retinal gliosis. Expression of Sox2, Nestin, and Pax2 is also shown in the idiopathic epiretinal membranes. Scale bar: 50 *µ*m.

Interestingly, the activated glial cells exhibit different cell morphologies in the iERMs. There are two main types of glial cells in the iERMs according to morphology: one is activated glial cells with long processes, and they appear to form a network by their processes (Figures 1(a)–1(e)); the other glial cell type forms dense cell aggregate, which are the resemblance of niERMs (Figures 2(f)–2(j)). These activated glial cells coexpress stem cell markers Sox2 and Nestin (Figures 1(a)–1(e) and 2(f)–2(j)).

In ILMs, there are a limited number of cells in comparison with the corresponding ERMs, and GFAP is also expressed in the composing cells (Figures 2(a)–2(c)). These glial cells also have long processes, and some of them coexpress progenitor cell markers: Sox2 and Nestin (Figures 2(d)–2(f)).

**Figure 3:**
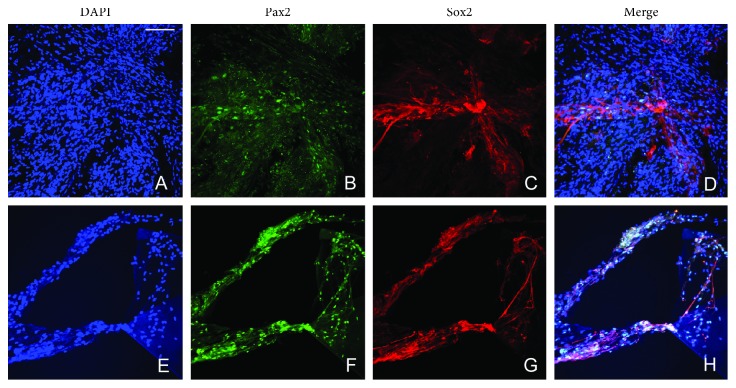
Presence of glial-like cells coexpresses Sox2 and Pax2 in the epiretinal membranes from patients with nonidiopathic retinal gliosis and idiopathic retinal gliosis. Expression of Sox2 and Pax2 are also shown in the nonidiopathic epiretinal membranes (from diabetic retinopathy) (a–d) and idiopathic epiretinal membranes (e–h). Scale bar: 20 *µ*m.

**Figure 4:**
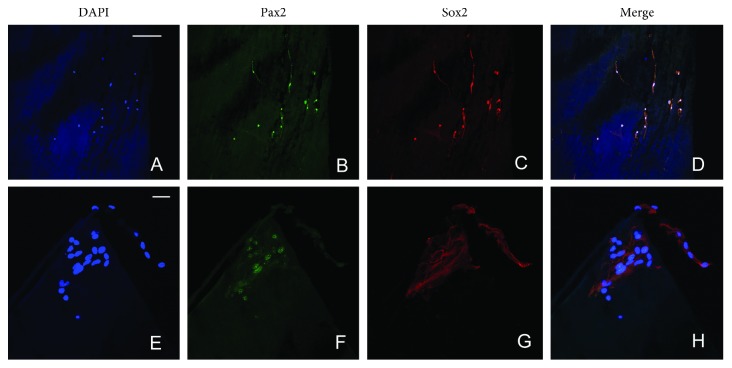
Presence of glial-like cells coexpresses Sox2 and Pax2 in the inner limiting membranes from patients with nonidiopathic retinal gliosis and idiopathic retinal gliosis. There are a limited number of cells in the ILMs. Expression of Sox2 and Pax2 is also shown in the inner limiting membranes from the patient with idiopathic retinal gliosis (a–d) and from the patient with PVR (e–h). Scale bar: 50 *µ*m.

Pax2 is a transcription factor which is necessary for glial cell differentiation. Sox2 and Pax2 are also expressed in both iERMs (Figures 3(a)–3(d)) and niERMs (Figures 3(e)–3(h)). In ILMs, some of the cells also express Sox2 and Pax2 (Figures 4(a)–4(h)).

**Figure 5:**
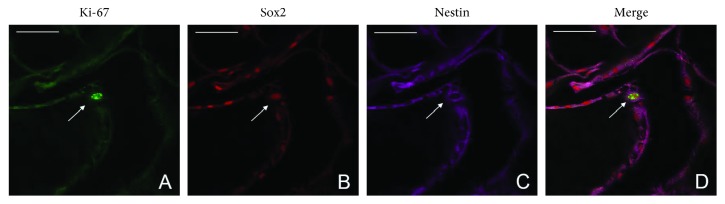
Expression of proliferation and progenitor cell markers epiretinal membranes. Ki-67 is expressed in the glial-like cells which coexpress with Sox2 and Nestin in ERMs (a–d). Scale bar: 50 *µ*m.

Ki-67, a marker of cell proliferation, was also found to be expressed in a few individual activated glial cells in the ERMs, and it appeared coexpressed with Sox2 (nuclear) and Nestin (cytoplasm), which indicates the proliferation potential of these progenitor cell types in the ERMs (Figure 5).

## 4. Discussion

This study investigated the morphological and progenitor cell markers' expression in idiopathic and nonidiopathic ERMs of different origins (e.g., associated with diabetic retinopathy, proliferative vitreoretinopathy, and uveitis). The first finding is the detection of two different types of glial cells found in ERMs based on their morphologies: one exhibited long branches and appeared to form local networks, as in iERMs and ILMs, while the other appeared more densely packed, as in niERMs. The second finding is the expression of markers of retinal progenitor cells (Sox2 and Nestin) and glial cell progenitor marker Pax2 in ERMs. Sox2 and Nestin were coexpressed in GFAP-positive glial cells in the ERMs, while Pax2 was expressed in almost all cell nuclei of GFAP-positive cells in the ERMs. These results suggest that activated glial cells are the dominant cell type in ERMs as well as in ILMs, and that these GFAP-positive cells are not pure glial cells, but glial-like cells due to coexpression of progenitor cell markers including Sox2, Pax2, and Nestin.

In the retina, Sox2 expression is required for proliferation and differentiation of retinal stem cells. Conditional ablation of Sox2 in retinal progenitor cells causes loss of competence in proliferation and differentiation [12]. In addition, Sox2 can maintain the state of retinal progenitor cells of postnatal retinal Müller cells [13]. In adult hippocampus, there are a number of GFAP-positive cells, called radial glia, which coexpress Sox2 and Nestin, and these cells are regarded as adult stem cells with ability to proliferate and differentiate into new neurons [14]. Nestin was also found to be expressed in the epiretinal membranes previously [9]. In human PVR eyes, the proliferating cells coexpressing neural stem cell markers (Nestin, Sox2, and Pax6) have been detected around the *ora serrata* region of the retina [10]. In our study, coexpression of the progenitor cell markers (Sox2, Pax2, and Nestin) in glial cells from ERMs suggests that these cells are not pure glial cells, but glial-like cells, which are likely a new cell type which could contribute to the proliferation and differentiation of glial cells in ERMs. Whether this is a plausible effect to have after surgery in regard to the technique of removing the ERMs/ILMs remains to be further studied with or without the use of the ILM flap technique and in correlation to the expression of progenitor cell markers in the fully or partially removed epiretinal tissue.

Pax2 is a regulator of the neuroglial cell fate determination in the optic nerve. If Pax2 is downregulated, the cells turn towards generating neurons, while overexpression of Pax2 triggers glial cell differentiation [15]. Our study found expression of the Ki-67 positive cell in the ERMs, although in a very limited number, suggesting that only a small number of progenitor cells can proliferate within the ERMs. They may also slowly proliferate and differentiate into a more glial cell phenotype. This result supports that ERMs may gradually develop and worsen. Together, these data demonstrate that glial cells expressing markers of progenitor cells exist in human ERMs, indicating similar intrinsic mechanism in the formation and development of retinal gliosis. The glial-like cells in the ERMs may have potential to differentiate into more glial cells under certain conditions *in vivo*, which require further studies.

Since the glial-like cells were found both in idiopathic and nonidiopathic retinal gliosis, it indicates they can play a similar role in both types of ERMs. In our mouse epiretinal gliosis model (unpublished), a number of activated glial cells resembling strong GFAP positivity were found in the retinal gliosis region induced by a *Bmpr1b* mutation; the GFAP-positive cells also expressed Pax2, Sox2, and Nestin. These data support the hypothesis that GFAP-positive precursors play a specific role in the pathogenesis of retinal gliosis.

Previous studies also found positive-GFAP staining in ERM specimens. GFAP is not exclusively positive in glial cells but also in cells of fibroblasts and extraretinal origin such as vitreous-derived cells hyalocytes [16, 17]. For example, coexpression of GFAP/CRALBP, GFAP/*α*-SMA, and *α*-SMA/CRALBP indicated that there was a transdifferentiation of Müller cells into fibroblasts and myofibroblasts [8]. GFAP-positive cells were positive for hyalocyte markers (anti-CD45, anti-CD64, and anti-CD34) which suggested a possible hyalocyte origin [18]. Our study showed that GFAP-positive cells in ERMs also expressed progenitor cell markers which indicate that these cells may not be fibroblasts or vitreous-derived cells. The origin of the glial cells coexpressing the markers of the progenitor cells remains unclear. One is that these cells may originate from the retinal Müller cells, which could have the properties of retinal progenitor cells; another is that glial cells in ERMs begin to transdifferentiate and acquire the fate of retinal progenitor cells, which may be due to local microenvironment change and presence of different factors. This should be confirmed in the future studies.

In conclusion, our study provides a new perspective of glial-like cells expressing progenitor cell markers in the pathogenesis of ERMs. The glial cells in the ERMs are unique, and some of these glial-like cells can proliferate and coexpress progenitor cell markers, such as Sox2, Pax2, and Nestin. This indicates that such cells may possess properties of stem-like cells, which can keep them self-renewed and/or differentiated towards more glial or neuroglial type of cells in the retina. Our study promotes further investigation on the stem cell properties of the glial cells found in retinal gliosis, with the aim to develop novel surgical and or pharmacological treatment modalities for retinal gliosis.

## Data Availability

The data used to support the findings of this study are available from the corresponding author upon request.

## Supplementary Materials

Supplementary Materials Supplementary Figure 1: negative controls of the stainings used throughout the study of epiretinal and inner limiting membranes. (A–H) Staining by secondary antibodies (antirabbit, antigoat, and antimouse) without primary antibody. The cells in ERMs and ILMs are shown not to stain for the secondary antibodies used. Scale bar: 50 *µ*m.
