# Supplementary figures and images for: Expression of Progenitor Cell Markers in the Glial-Like Cells of Epiretinal Membranes of Different Origins
Source: J Ophthalmol. 2018 Dec 27;2018:7096326. doi: 10.1155/2018/7096326 (PMC6327511; doi:10.1155/2018/7096326)

## Supplemental Fig.1

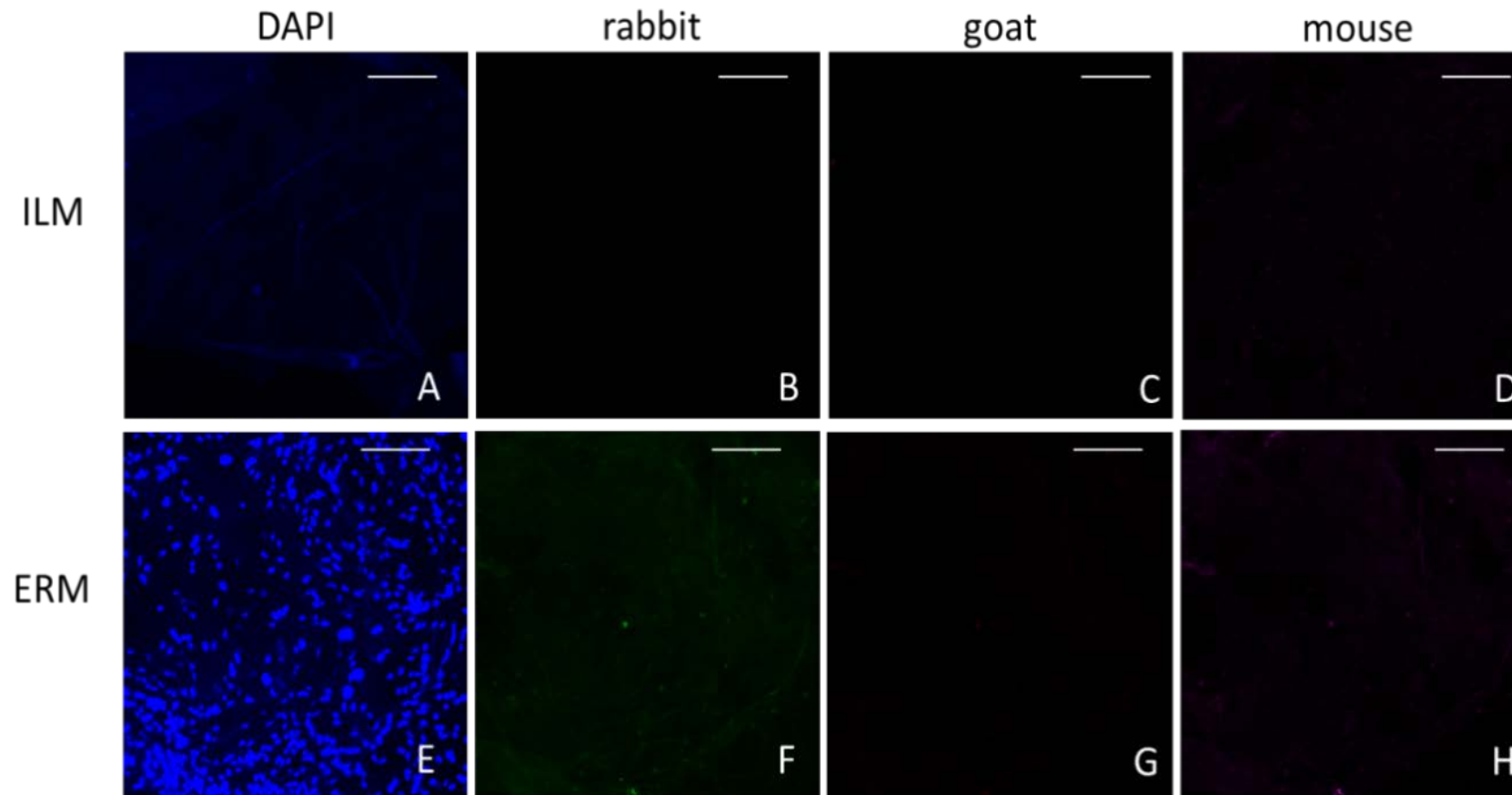

Supplement: Supplementary Materials — Supplementary Figure 1: negative controls of the stainings used throughout the study of epiretinal and inner limiting membranes. (A–H) Staining by secondary antibodies (antirabbit, antigoat, and antimouse) without primary antibody. The cells in ERMs and ILMs are shown not to stain for the secondary antibodies used. Scale bar: 50 µm. [file 7096326.f1.pdf]
